# Survival analysis of patients with advanced non-small cell lung cancer receiving EGFR-TKI treatment of Yunnan in southwestern China: a real-world study

**DOI:** 10.3389/fonc.2023.1156647

**Published:** 2023-10-10

**Authors:** Yanping Lin, Long Chen, Rong Li, Xin Liu, Quan Li, Jingjing Cai, Yaxi Du, Guangqiang Zhao, Xiaoxiong Wang, Zhenghai Shen, Yedan Liao, Yang Chen, Lin Xie, Yongchun Zhou, Yunchao Huang

**Affiliations:** ^1^ Department of Digestive Neoplasms, The Third Affiliated Hospital of Kunming Medical University (Yunnan Tumor Hospital), Kunming, China; ^2^ PET/CT Center, The Third Affiliated Hospital of Kunming Medical University (Yunnan Tumor Hospital), Kunming, China; ^3^ Molecular Diagnosis Center of Yunnan Cancer Hospital, The Third Affiliated Hospital of Kunming Medical University (Yunnan Tumor Hospital), Kunming, China; ^4^ Yunnan Provincial Key Laboratory of Lung cancer, The Third Affiliated Hospital of Kunming Medical University (Yunnan Tumor Hospital), Kunming, China; ^5^ Department of Thoracic Surgery I, The Third Affiliated Hospital of Kunming Medical University (Yunnan Tumor Hospital), Kunming, China; ^6^ Department of Chronic Disease Management, Yunnan Center for Disease and Prevention and Control, Kunming, China

**Keywords:** lung cancer, non-small cell lung cancer, EGFR, TKI, uncommon mutation, Yunnan, Xuanwei

## Abstract

**Importance:**

Patients with EGFR mutations who have advanced-stage non-small cell lung cancer (NSCLC) already receive tyrosine kinase inhibitors (TKIs) as the standard first-line therapy. Notably, Yunnan is a regional high incidence area of lung cancer in the highlands with a high rate of rare EGFR mutations. Overall, lung cancer patients in Xuanwei may present a distinct subgroup globally. Recent studies suggested that the NSCLC cohort in Xuanwei harbored a significantly higher uncommon mutation rate. However, little was known about the clinicopathological features and treatment efficacy of EGFR-TKI in Yunnan NSCLC patients.

**Objective:**

This study aimed to investigate the clinical impact of histologic type on the survival outcomes of patients with stage IIIB and IV NSCLC receiving EGFR-TKI treatment of Yunnan in southwestern China.

**Methods:**

In this retrospective study, we enrolled advanced NSCLC patients (IIIB-IV) with EGFR mutations who were first diagnosed and treated at Yunnan Cancer hospital from January 2016 to December 2019. Sociodemographics, lifestyle, survival, and clinicopathological characteristics of the patients were collected. The Kaplan-Meier method was used to assess the OS and PFS of patients. An analysis of prognostic factors was conducted using Cox regression.

**Results:**

A total of 468 eligible patients were included. The median progression-free survival (PFS) and overall survival(OS) were 11.30(95% CI, 10.12-12.48) months and 30.30(95% CI, 26.24-34.36) months. Based on survival analysis among all the patients,females(HR=0.815;95% CI:0.671-0.989; *P*=0.017), Xuanwei origin (HR=0.776; 95% CI: 0.609-0.989; *P*=0.040), sample types(HR=0.780; 95% CI: 0.642-0.947; *P*=0.012) had a longer PFS. Multivariable analysis showed that only the sample type was an independent factor on median PFS with EGFR-TKI therapy. Patients less than 60 years old (HR=1.433; 95% CI:1.134-1.812, *P*=0.003)had better OS, but objectives with BMI≥24kg/m^2^(HR=0.653; 95% CI: 0.500-0.864; *P*=0.002), females(HR=0.776; 95% CI:0.613-0.982; *P*=0.035)and patients with tissue sample type (HR=0.760; 95% CI:0.600-.0961; *P*=0.022) had better OS. Notably, subgroup analysis of our study also found that PFS was significantly better in patients with G719X, L861Q, S768I, G719X+L861Q, and G719X+S768I in Xuanwei than classical mutation ones, including 19-Del and L858R (median 22.7 vs. 12.0 months, HR=0.523, *P*=0.010), while PFS was inferior in patients with rare mutations of EGFR in non-Xuanwei than the classical mutation ones (median 5.10 vs. 11.10 months, HR=1.760, *P*=0.015).

**Conclusion:**

NSCLC patients in Yunnan displayed a unique EGFR mutation profile, especially a higher prevalence of EGFR uncommon and compound mutations subtype. This study indicates prognostic factors of NSCLC treated with EGFR-TKI in Yunan and Xuanwei. This study will provide new clinical evidence for EGFR-TKI-targeted therapy in patients with rare EGFR mutations in China and worldwide. More researchs were needed for NSCLC EGFR-TKI therapy and medical insurance policy-making in Yunnan, Xuanwei area and uncommon especially.

## Introduction

The first and most common cause of cancer death worldwide is lung cancer, and NSCLC accounts for 80% to 85% of all lung cancer deaths (1). When a patient is initially diagnosed, they may have advanced stages, following the claim that epidermal growth factor receptor mutations drive NSCLC (2). Patients with EGFR mutations who have advanced-stage NSCLC already receive tyrosine kinase inhibitors (TKIs) as the standard first-line therapy (3).

However, patient groups encountered in clinical practice do not meet the stringent inclusion criteria required for participation in clinical trials. Therefore, the effectiveness of EGFR-TKIs in patients treated in the natural world setting remains unclear. Notably, Yunnan is a regional high incidence area of lung cancer in the highlands with a high rate of rare EGFR mutations. Overall, lung cancer patients in Xuanwei may present a distinct subgroup globally (4, 5). Recent studies suggested that the NSCLC cohort in Xuanwei harbored a significantly higher uncommon complexed mutation rate. Little was known about the clinicopathological features and treatment efficacy of EGFR-TKI in Yunnan NSCLC patients (6). Here, we explored the effectiveness of TKI in Yunan-advanced NSCLC patients with EGFR mutation. To the best of our knowledge, this study is currently the first real-world study related to EGFR-TKI for NSCLC in Yunnan.

## Materials and methods

### Patients selection

For this retrospective cohort study, advanced NSCLC patients treated with TKI from January 2016 to August 2019 in the Molecular Diagnostic Center of Yunnan Cancer Hospital were enrolled. Inclusion criteria: ①First diagnosis and treatment in the hospital; ②Local residents; ③Age≥18 years; ④Clinical stage IIIB or IV; ⑤With EGFR mutation; ⑥Treatment with EGFR TKIs. Exclusion criteria: ①None first-line treatment; ②Cases with medical records were incomplete.

The medical records and EGFR genotype data of 3007 NSCLC patients were retrospectively collected from our hospital from 16 sites, including all of the sites in Yunnan. Clinical data were collected from the medical records of each patient. This included patients characteristics(date of NSCLC diagnosis, sex, age, histological diagnosis, clinical staging, distant metastasis organ, smoking history, drinking history, and type of EGFR mutation); survival data (status as of the end of April 2022, date of death or date of the last follow-up).

### Data collection

Formalin-fixed paraffin-embedded (FFPE) tumor tissues, fine-needle aspiration and core needle biopsies, pleural effusion cells, and plasma samples were used to detect mutations. Genomic DNA and total RNA were extracted from FFPE samples using the AmoyDx FFPE DNA/RNA extraction kit (Amoy Diagnostics, Xiamen, China) following the manufacturer’s protocols. For other types of models, an AmoyDx Tissue DNA/RNA extraction kit (Amoy Diagnostics) was used. An Amplification Refractory Mutation System Polymerase Chain Reaction (ARMS-PCR) and a Mutation Detection Kit (Amoy Diagnostics) were used to detect the EGFR mutations.

The clinical stage was evaluated according to the 8th edition of the American Joint Committee on Cancer (AJCC) tumor-node-metastasis (TNM) classification system. The tumor response to TKI was assessed based on the Response Evaluation Criteria in Solid Tumors (RECIST) version 1.1. The primary endpoints of this study were PFS, which was defined as the time from initiating EGFR-TKI treatment to the date of disease progression or the last follow-up. Overall survival (OS) was defined as the interval from the first dose of first-line treatment until the date of death. Time to disease progression and survival for all patients was obtained by the active follow-up (telephone follow-up) and passive follow-up (case database review and tumor registry database matching) for the study subjects with a follow-up deadline of April 30, 2022.

### Statistical analysis

Descriptive statistics presented patients’ baseline characteristics. And the data were presented as a percentage for dichotomous variables and analyzed using a chi-square test or Fisher’s exact test. Kaplan-Meier method was used to calculate the curves for PFS between groups. The Cox proportional hazards regression model was used to evaluate the impact of collected variables on PFS. The log-rank test determined significant differences. A two-tailed with *a P* value less than 0.05 was considered statistically significant. Statistical analyses were performed using SPSS^®^ software, version 20.0 (IBM Corp, Armonk, NY, USA).

## Results

**Figure 1:**
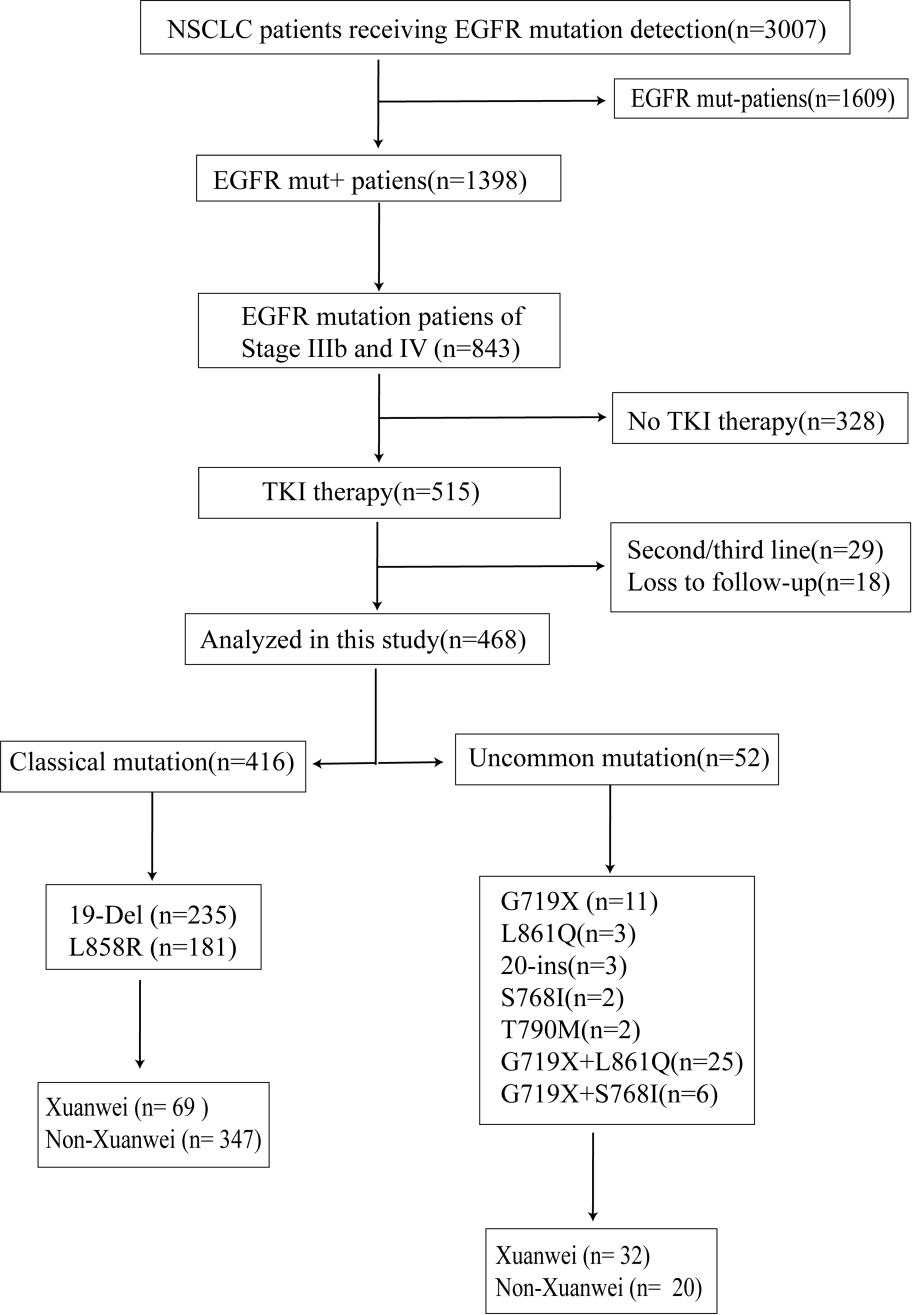
Flowchart of the study.

Between 1 January 2016 and 30 December 2019, 3007 patients received EGFR mutation detection, and 1398 patients were mutant. A total of 515(61.09%) patients with stage IIIB and IV received EGFR TKI therapy, but 18 cases lost follow-up, and 29 objectives were the non-first line to receive TKI. Treatment and survival details of 468 patients were enrolled from 16 sites, including all of the sites in Yunnan, **Figure S1**. Among these 468 patients who received TKI therapy, over half, 235(50.21%) of patients had tumors with an EGFR 19-Del mutation, and 181 (38.68%) had the L858R mutation. A total of 52 patients (11.11%) had uncommon mutations and these are detailed in **Figure 1**, included G719X(n=11), L861Q(n=3), 20-ins(n=3), S768I(n=2), G719X+L861Q(n=25), G719X+S768I(n=6), T790M(n=2). The patient’s identification flow charts are illustrated in **Figure 1**. The clinicopathological characteristics, including sex, age at diagnosis, smoking history, staging, ethnic, area, smoking history, drinking history, type of specimen, clinical stage, drugs and type of EGFR mutation, are listed in **Table 1**.

**Table 1:** Baseline factors of patients receiving tyrosine kinase inhibitor (TKI) treatment.

Characterristics	N	%
Gender		
Male	203	21.58
Female	265	78.42
Ages		
<60	271	57.91
≥60	197	42.09
Nationality		
Han	386	82.48
Minority	82	17.52
Area ^a^		
Xuanwei	101	21.58
Non-xuanwei	367	78.42
Smoking history		
Smoker	142	30.34
Never-smoker	326	69.66
Drinking history		
Yes	110	23.50
No	358	76.50
Types of spicimen		
Tissue	266	56.84
Peripheral blood and pleural effusion	202	43.16
Clinical Stage		
IIIb	47	10.04
IV	421	89.96
Drugs		
Gefitinib	275	58.76
Icotinib	163	34.83
Erlotinib	6	1.28
Afatinib	10	2.14
Osimertinib	5	1.07
unknown	9	1.92
Types of mutation		
Classical mutation	416	88.89
Uncommon mutation	52	11.11

a: Using the area classification of the National Bureau of Statistics in Yunnan, we categorized each patient’s place of residence into Xuanwei and Non-Xuanwei county.

**Figure 2:**
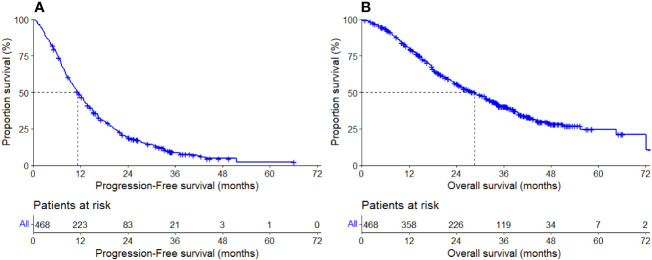
PFS and OS in the overall group. **(A)** PFS of overall group. **(B)** OS of overall group.

The median duration of follow-up was 38.29 months(95% CI, 37.31-39.27m). At the end of follow-up, 415 patients (88.68%) had disease progression or died (progressed: n=367, 78.42%; died: n=48, 10.25%), and 53 (11.32%) were censored. The median PFS was 11.30 months(95% CI,10.12-12.48m), **Figure 2A**.

**Figure 3:**
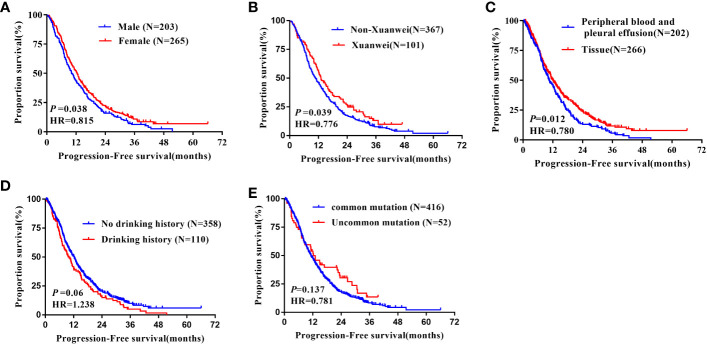
Kaplan-Meier curves showing the PFS of the sequential EGFR-TKI treatment in patients with distinct clinical subgroups. **(A)** Genders; **(B)** Region; **(C)** Types of genetic testing specimens; **(D)** Drinking history; **(E)** Type of EGFR mutation.

The median PFS of females (12.50 months, 95%CI: 11.21-13.79m) was longer than males(12.50 vs.10.20 months, hazard ratio = 0.815, 95% CI 0.671 to 0.989, *P* = 0.017, **Figure 3A**). Again, the median PFS was longer in Xuanwei origins compared to non-Xuanwei origins(13.00 vs. 10.70months, hazard ratio =0.776, 95% CI 0.609 to 0.989, *P*=0.040, **Figure 3B**). PFS benefit longer for tissue samples patients(12.00 vs.10.50 months, hazard ratio=0.780, 95% CI 0.642 to 0.947, *P* =0.012, **Figure 3C**). No statistical difference in PFS between patients with classical EGFR mutations and those with rare mutations, *P*=0.135, **Figure 3E**. PFS analysis by age, nationality, BMI, smoking status, hypertension history, diabetes history, lung cancer family history, clinical stage, brain metastasis, and types of TKI drugs revealed differences without statistical significance(*P*>0.05), **Table 2** and **Figure 3**.

**Table 2:** Relationship of PFS and OS with clinical characteristics.

Parameters	Numberrr	Mean PFS(95%CI, month) PFSPFSPFS(95%CI,month)	Chi-square	*P value valuevalue*	Mean OS(95%CI,month)	Chi-square	*P value*
**Gender**			4.324	0.038		9.171	0.002
Male	203	10.20 (8.58-11.83)			21.90(17.58-26.22)		
Female	265	12.50(11.21-13.79)			32.50(27.84-37.16)		
**Ages**			2.113	0.146		14.751	<0.001
<60	271	12.00(10.81-13.19)			34.60(29.00-40.20)		
≥60	197	10.20( 7.97-12.43)			21.90(17.58-26.22)		
**Nationality**			3.607	0.058		0.097	0.755
Han	386	12.20(10.84-13.56)			28.80(24.85-32.75)		
Minority	82	9.10(7.41-10.79)			27.50(18.15-36.85)		
**Region**			4.267	0.039		2.305	0.130
Xuanwei	101	13.00(10.31-15.69)			34.80(27.07-42.53)		
Non-xuanwei	367	10.70( 9.32-12.08)			27.00(23.01-30.99)		
**BMI,kg/m^2^**			0.424	0.51		9.899	0.002
<24kg/m^2^	322	11.50(10.10-12.90)			24.60(20.87-28.33)		
≥24kg/m^2^	146	11.30(9.13-13.47)			39.50(32.27-46.73)		
**Smoking history**			0.503	0.478		2.631	0.106
Smoker	142	10.80(8.45-13.15)			25.00(19.36-30.64)		
Never-smoker	326	12.00(10.51-13.49)			30.30(25.52-35.08)		
**Drinking history**			3.509	0.060		2.253	0.135
Yes	110	10.00(7.92-12.08)			25.10(16.42-33.78)		
No	358	12.20(10.80-13.60)			28.90(24.56-33.25)		
**Hypertension history**			0.236	0.627		0.025	0.873
Yes	92	12.20(9.12-15.28)			28.90(20.60-37.21)		
No	376	11.30(10.05-12.55)			27.60(23.67-31.53)		
**Diabetes history**			2.584	0.108		0.857	0.355
Yes	17	10.10(6.97-13.23)			19.70(2.20-37.20)		
No	451	11.50(10.26-12.74)			28.70(24.96-32.45)		
**Lung cancer family history**			2.059	0.151		3.490	0.062
Yes	12	6.80(4.93-8.67)			21.50(11.03-31.07)		
No	456	11.30(10.09-12.51)			28.80(24.78-32.83)		
**Types of specimen**			6.371	0.012		5.292	0.022
Tissue	266	12.00(10.47-13.53)			31.50(27.45-35.55)		
Peripheral blood and pleural effusion	202	10.50(8.68-12.32)			25.10(20.61-29.60)		
**Clinical Stage**			0.004	0.948		1.548	0.213
IIIb	47	11.20(7.74-14.66)			39.50(31.82-47.18)		
IV	421	11.30(10.06-12.54)			27.50(23.80-31.20)		
**Brain metastasis**			2.048	0.152		2.234	0.135
Yes	138	10.10(8.29-11.91)			25.00(20.68-29.32)		
No	330	12.10(10.67-13.53)			31.40(26.46-36.34)		
Drugs*			6.098	0.297		6.312	0.277
Gefitinib	275	12.00(10.57-13.43)			28.80(23.40-34.20)		
Icotinib	140	12.50(9.86-15.14)			27.60(21.98-31.22)		
Erlotinib	5	10.00(8.25-11.75)			25.4		
Afatinib	6	23.50(3.22-43.78)			28.60(18.00-39.20)		
Osimertinib	5	8.70(5.48-11.92)			31.90(7.41-56.39)		
unknown	8	12.20(2.87-21.53)			18.20(4.45-31.95)		
**Types of mutation**			2.234	0.137		0.517	0.473
classical mutation	416	11.20(9.97-12.43)			27.50(23.79-31.23)		
Uncommon mutation	52	12.20(7.96-16.44)			33.80(27.21-40.39)		

Drugs*: There were 416 patients with common mutations of EGFR, of which 245 (58.89%) were on oral gefitinib, 152 (36.54%) on oral icotinib, 5 on oral osimertinib, 4 on oral erlotinib, 2 on oral afatinib, and 8 on specific no drug not known.

Multivariate analysis indicated that EGFR gene test sample type was an independent factor affecting PFS in patients treated with EGFR-TKI (HR=0.814, 95%CI: 0.669-0.991; *P*=0.040). However, gender and regional distribution of patients (Xuanwei origins versus non-Xuanwei origins) were not independent factors affecting PFS in patients treated with EGFR-TKI, **Table 3**.

**Table 3:** Progress free survival and overall survival: univariate and multivariate analysis.

Parameters	PFS				OS			
	Univariate		Multivariate		Univariate		Multivariate	
	HR(95%CI)	*P value*	HR(95%CI)	*P value*	HR(95%CI)	*P value*	HR(95%CI)	*P value*
Gender(Female)	0.815(0.671-0.989)	**0.017**	0.824(0.679-1.001)	0.051	0.776(0.613-0.982)	**0.035**	0.778(0.614-0.986)	**0.038**
Age(≥60)	1.155(0.950-1.404)	0.148			1.433(1.134-1.812)	**0.003**	1.391(1.099-1.760)	**0.006**
Nationality(Minority)	1.270(0.991-1.628)	0.059			1.052(0.766-1.443)	0.755		
Region(Xuanwei)	0.776(0.609-0.989)	**0.040**	0.886(0.685-1.147)	0.360	0.791(0.583-1.072)	0.130		
BMI(≥24kg/m^2^)	0.933(0.757-1.150)	0.517			0.653(0.500-0.864)	**0.002**	0.658(0.503-0.861)	**0.002**
Smoking history(Smoker)	1.079(0.874-1.330)	0.480			1.228(0.957-1.575)	0.106		
Drinking history(Yes)	1.238(0.989-1550)	0.060			1.225(0.939-1.599)	0.135		
Hypertension history(Yes)	0.942(0.741-1.198)	0.628			0.977(0.731-1.305)	0.873		
Diabetes history(Yes)	1.502(0.910-2.280)	0.112			1.346(0.715-2.531)	0.357		
Lung cancer family history(Yes)	1.517(0.853-2.698)	0.156			1.812(0.962-3.416)	0.066		
Types of specimen(Tissue)	0.780(0.642-0.947)	**0.012**	0.814(0.669-0.991)	**0.040**	0.760(0.600-.0961)	**0.022**	0.787(0.622-0.997)	**0.047**
Clinical Stage(IV)	0.997(0.921-1.080)	0.948			1.071(0.961-1.194)	0.216		
Brain metastasis(Yes)	1.164(0.945-1.434)	0.154			1.211(0.941-1.558)	0.136		
Types of mutation(Uncommon mutation)	0.781(0.563-1.082)	0.137			0.867(0.586-1.281)	0.473		

There were 52 patients with rare mutations, of which 30 (57.69%) were on oral gefitinib, 11 (21.15%) on oral icotinib, 9 (17.31%) on oral afatinib, and 1 patient each on oral erlotinib and ositinib.

Bold values provided in **Tables 3**–**7** represents a p-value of less than 0.05, which is statistically different.

In addition, the median OS was 30.30 months (95% CI, 26.24-34.36m, **Figure 2B**). During follow-up for OS, 280 (59.83%) patients died, and 188 patients were censored (40.17%). Reasons for censoring included: regular end of study (n=158, 84.04%); lost to follow-up (n=20,10.64%); patient’s wish (n=10, 5.32%).

**Figure 4:**
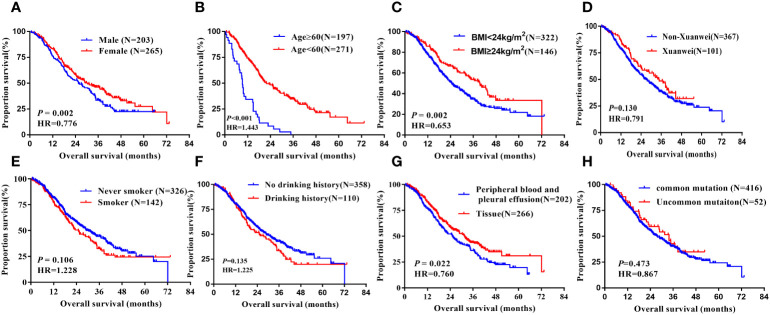
Kaplan-Meier curves showing the OS of the sequential EGFR-TKI treatment in patients with distinct clinical subgroups. **(A)** Genders; **(B)** Ages; **(C)** BMI; **(D)** Country; **(E)** Smoking history; **(F)** Drinking history; **(G)** Types of genetic testing specimens; **(H)** Type of EGFR mutation.

In the analysis of overall survival, there was a significant difference with gender, age, BMI, and the specimen types of the EGFR mutation test. Median OS was longer in females versus males (32.50 vs.21.90 months, hazard ratio =0.776, 95% CI 0.613 to 0.982, *P* =0.035, **Figure 4A**). Significantly longer OS was noted in patients with less than 60 years old group than more than 60 ones(34.60 vs21.90 months, hazard ratio=1.433, 95% CI 1.134 to 1.812, *P* =0.003, **Figure 4B**), and in those patients whose BMI more than 24kg/m^2^ had longer OS than others (39.50 vs.24.60 months, hazard ratio=0.653, 95% CI 0.500 to 0.864, *P* =0.002, **Figure 4C**). What’s more, in terms of the sample types, tissue samples tested for PFS were longer than other sample types (31.50 vs.25.10 months, hazard ratio=0.760, 95% CI 0.600 to 0.961, *P* =0.022, **Figure 4G**). However, factors such as ethnicity, smoking history, alcohol consumption, hypertension, diabetes, family history of lung cancer, disease stage, EGFR mutation type, and EGFR-TKI drug type were not associated with OS of patients after EGFR-TKI treatment, not statistically significant (*P*>0.05, **Figure 4D–F, H**), **Table 2**.

In multivariate analyses using multiple Cox proportional hazards models, we observed that gender(HR=0.778, 95% CI:0.614-0.986, *P=*0.038), age(HR=1.391, 95% CI:1.099-1.760, *P=*0.006), BMI (HR=0.658, 95% CI:0.503-0.861, *P*=0.002), types of specimen for EGFR mutation test (HR=0.787, 95% CI:0.622-0.997, *P*=0.047) were the independent prognostic factors for OS, **Table 3**.

Our group has long been engaged in research on the etiology, prevention, and treatment of lung cancer in Xuanwei, Yunnan Province (7, 8). Previous studies (5, 9)found that the incidence and mortality rates of lung cancer in Xuanwei are significantly higher than in other regions. It is characterized by a high rate of rare mutations and compound mutations in EGFR. Meanwhile, univariate analysis in this study suggested that there is a significant difference in PFS between Xuanwei and non-Xuanwei patients with non-small cell lung cancer after first-line treatment with EGFR-TKI, so we performed a subgroup analysis to explore the possible causes of the difference.

### Subgroup analysis

**Figure 5:**
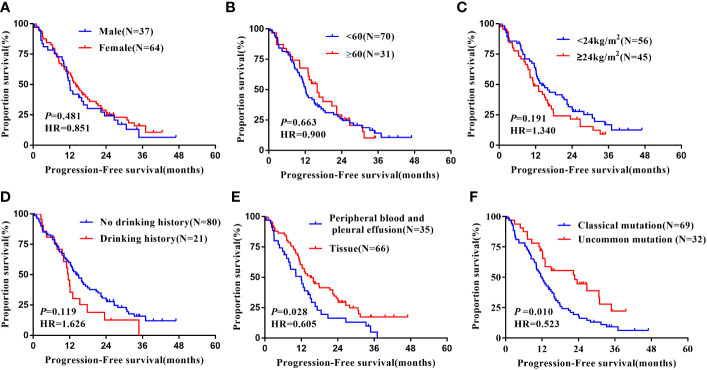
Subgroup analysis in Xuanwei lung cancer patients of mPFS. **(A)** Genders; **(B)** Ages; **(C)** BMI; **(D)** Drinking history; **(E)** Types of genetic testing specimens; **(F)** Type of EGFR mutation.

**Figure 6:**
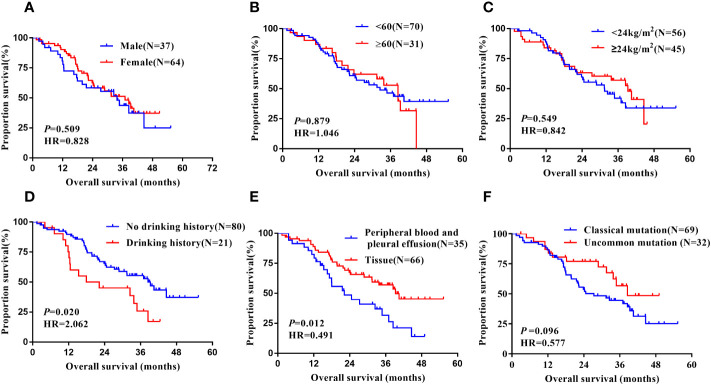
Subgroup analysis in Xuanwei lung cancer patients of mOS. **(A)** Genders; **(B)** Ages; **(C)** BMI; **(D)** Drinking history; **(E)** Types of genetic testing specimens; **(F)** Type of EGFR mutation.

Notably, subgroup analysis based on the patients’ region of this study, **Table 4**, also found that, in the Xuanwei group, the PFS of patients with uncommon EGFR mutations was significantly better than classical mutations patients (median 22.70 vs. 12.00 months, HR=0.523, 95% CI 0.318 to 0.862, *P* =0.011, **Figure 5F**). Similarly, we found that the OS was longer in uncommon EGFR mutations vs. common ones (median 38.50 vs. 27.30 months, HR=0.577, 95% CI 0.302 to 1.103, *P*=0.096, **Figure 6F**).

**Table 4:** Progress free survival and overall survival: univariate analysis of Xuanwei county lung cancer patients.

Parameters	Number	PFS(95%CI,month)	Chi-square	*P*	OS(95%CI,month)	Chi-square	*P*
**Gender**			0.500	0.479		0.439	0.508
Male	37	12.10(10.79-13.41)			33.80(22.91-44.69)		
Female	64	14.00(11.07-16.93)			37.00(26.81-47.20)		
**Ages**			0.191	0.622		0.023	0.879
<60	70	12.10(10.62-13.58)			32.50(21.65-43.35)		
≥60	31	15.90(11.87-19.93)			38.50(30.26-46.74)		
**Nationality**			2.387	0.122		0.408	0.523
Han	94	13.00(10.32-15.68)			34.80(28.49-41.12)		
Minority	7	6.4(2.29-10.51)			21.50(NR)		
**BMI,kg/m^2^**			1.735	0.188		0.362	0.547
<24kg/m^2^	56	14.00(9.75-18.25)			31.50(20.73-42.28)		
≥24kg/m^2^	45	11.30(10.31-15.69)			39.30(31.91-56.70)		
**Smoking history**			0.947	0.33		3.189	0.074
Smoker	29	12.00(10.59-13.41)			22.20(4.18-40.22)		
Never-smoker	72	14.00(10.67-17.33)			38.50(29.46-47.54)		
**Drinking history**			2.485	0.115		5.713	**0.017**
Yes	21	11.50(10.06-12.94)			22.20(0.00-44.981)		
No	80	14.20(11.08-17.32)			39.30(29.24-49.36)		
**Hypertension history**			3.149	0.076		1.843	0.175
Yes	10	10.20(6.58-13.82)			21.50(9.16-33.84)		
No	91	13.30(10.19-16.42)			37.00(30.19-43.81)		
**Diabetes history**			1.525	0.219		0.729	0.393
Yes	5	13.00(10.00-16.00)			NR		
No	96	13.00(6.99-19.01)			33.80(26.47-41.14)		
**Lung cancer family history**			2.068	0.150		1.82	0.177
Yes	5	13.00(10.54-15.46)			21.50(13.13-29.87)		
No	96	7.10(0.00-18.48)			37.00(29.99-44.01)		
**Types of specimen**			4.983	**0.026**		6.603	**0.010**
Tissue	66	15.00(11.39-18.61)			39.30(NR)		
Peripheral blood and pleural effusion	35	12.00(6.80-17.20)			22.20(11.39-33.01)		
**Clinical Stage**			0.003	0.959		2.036	0.154
IIIb	14	14.00(6.65-21.35)			40.50(10.95-70.05)		
IV	87	13.00(10.07-15.93)			32.50(23.45-41.55)		
**Brain metastasis**			0.773	0.379		0.875	0.350
Yes	70	12.00(10.58-13.42)			31.50(16.41-46.60)		
No	31	14.20(10.83-17.57)			38.50(29.28-47.72)		
**Drugs**			2.557	0.279		1.926	0.382
Gefitinib	63	15.90(12.02-19.78)			33.80(21.66-45.94)		
Icotinib	31	10.20(7.26-13.15)			32.50(10.02-54.98)		
Afatinib	7	23.50(12.73-34.27)			31.50(0.00-65.97)		
**Types of mutation**			6.713	**0.010**		2.847	0.092
classical mutation	69	12.00(9.29-14.71)			27.30(15.60-39.00)		
Uncommon mutation	32	22.70(9.03-36.37)			38.50(NR)		

Bold values provided in **Tables 3**–**7** represents a p-value of less than 0.05, which is statistically different.

In the Xuanwei lung cancer patients subgroup, OS was significantly prolonged in patients with no history of alcohol consumption compared to those with a history of alcohol consumption(median 39.30 vs. 22.20 months, HR=2.062, 95% CI 1.123 to 3.785, *P*=0.020, **Figure 5D**). In addition, the study also showed that patients with tissue samples had better PFS(median 15.00 vs. 12.00 months, HR=0.523, 95% CI 0.318 to 0.862, *P*=0.011, **Figure 5E**) and OS (median 39.30 vs. 22.20 months, HR=0.491, 95% CI 0.282 to 0.855, *P*=0.012, **Figure 6E**) than other sample ones. In contrast, gender, age, ethnicity, smoking history, and family history of lung cancer were not associated with PFS and OS of TKI therapy in patients with non-small cell lung cancer in this region (P>0.05, **Figures 5A–C**, **6A–D**). Multifactorial analysis, **Table 5**, EGFR mutation type, which is divided into classical and uncommon mutation types, was an independent factor influencing PFS of TKI treatment in patients in Xuanwei (HR=0.523,95% CI 0.318-0.862, *P*=0.011). In addition the specimen types (HR=0.520, 95% CI 0.297-0.909, *P*=0.022) and history of alcohol consumption (HR=1.911, 95% CI 1.036-3.524, *P*=0.038) were independent influencing factors for OS.

**Table 5:** Progress free survival and overall survival: univariate and multivariate analysis in Xuanwei lung cancer subgroup.

Parameters	PFS				OS			
	Univariate		Multivariate		Univariate		Multivariate	
	HR(95%CI)	*P value*	HR(95%CI)	*P value*	HR(95%CI)	*P value*	HR(95%CI)	*P value*
Gender(Female)	0.851(0.544-1.332)	0.481			0.828(0.474-1.448)	0.509		
Age(≥60)	0.900(0.561-1.445)	0.663			1.046(0.584-1.876)	0.879		
Nationality(Minority)	1.827(0.839-3.980)	0.129			1.462(0.452-4.728)	0.526		
BMI(≥24kg/m^2^)	1.340(0.864-2.078)	0.191			0.842(0.481-1.476)	0.549		
Smoking history(Smoker)	1.266(0.786-2.040)	0.333			1.668(0.945-2.946)	0.078		
Drinking history(Yes)	1.526(0.898-2.593)	0.119			2.062(1.123-3.785)	**0.020**	1.911(1.036-3.524)	**0.038**
Hypertension history(Yes)	1.881(0.924-3.83)	0.082			1.793(0.761-4.220)	0.182		
Diabetes history(Yes)	1.766(0.703-4.435)	0.226			0.433(0.060-3.144)	0.408		
Lung cancer family history(Yes)	1.920(0.774-4.763)	0.159			2.005(0.714-5.631)	0.187		
Types of specimen(Tissue)	0.605(0.387-0.946)	**0.028**	0.708(0.445-1.127)	0.145	0.491(0.282-0.855)	**0.012**	0.520(0.297-0.909)	**0.022**
Clinical Stage(IV)	0.996(0.843-1.175)	0.959			1.199(0.929-1.549)	0.164		
Brain metastasis(Yes)	1.230(0.774-1.956)	0.382			1.325(0.732-2.399)	0.352		
Types of mutation(Uncommon mutation)	0.523(0.318-0.862)	**0.011**	0.523(0.318-0.862)	**0.011**	0.577(0.302-1.103)	0.096		

Bold values provided in **Tables 3**–**7** represents a p-value of less than 0.05, which is statistically different.

**Figure 7:**
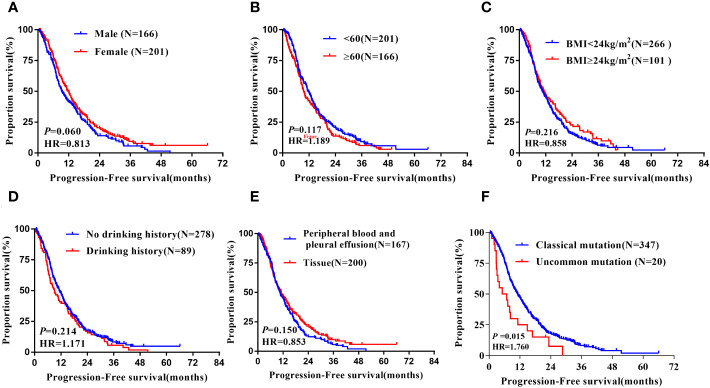
Subgroup analysis in Non-Xuanwei lung cancer patients of mPFS. **(A)** Genders; **(B)** Ages; **(C)** BMI; **(D)** Drinking history; **(E)** Types of genetic testing specimens; **(F)** Type of EGFR mutation.

**Figure 8:**
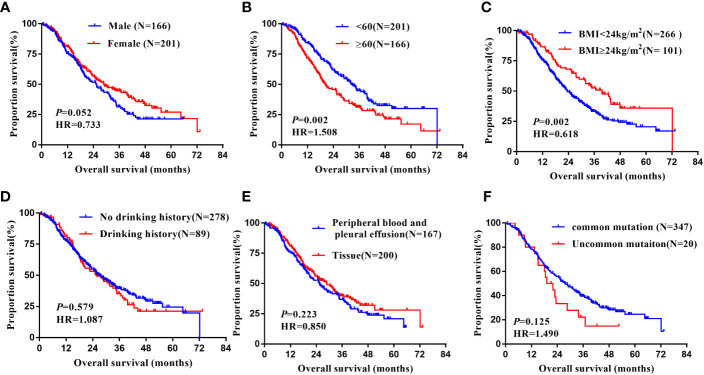
Subgroup analysis in Non-Xuanwei lung cancer patients of mOS. **(A)** Genders; **(B)** Ages; **(C)** BMI; **(D)** Drinking history; **(E)** Types of genetic testing specimens; **(F)** Type of EGFR mutation.

In the non-Xuanwei group, **Table 6**, the mPFS of patients with uncommon EGFR mutations in non-Xuanwei was significantly lower than classical mutation ones(median 5.10 vs.11.10 months, HR=1.760, 95% CI 1.106 to 2.800, *P*=0.017, **Figure 7F**). Similarly, we found that the OS was longer in non-Xuanwei origins with uncommon mutation patients vs. Classical mutation ones(median 19.10 vs. 28.80 months, HR=1.490,95% CI 0.895 to 2.479, *P*=0.125). Still, no statistical difference was reached, **Figure 8F**. In the subgroup of patients from non-Xuanwei origins, the univariate analysis suggested that age was associated with patient OS, and OS in the <60 years age group was significantly better than that in the≥60 years age group (median 32.70 vs. 20.20 months, HR=1.508, 95% CI 1.164 to 1.955, *P*=0.002, **Figure 8B**). OS was significantly better in patients with BMI≥24 kg/m^2^ than in patients with BMI<24 kg/m2(median 39.50 vs. 23.30 months, HR=0.618, 95% CI 0.453 to 0.843, *P*=0.002, **Figure 8C**, while gender, ethnicity, history of smoking, history of hypertension, history of diabetes mellitus, family history of lung cancer, sample types, and clinical stage were not associated with TKI efficacy in NSCLC patients in this region (P>0.05, **Figures 7A–E**, **8A, D, E**). Multifactorial analysis showed that in **Table 7**, mutation type was an independent factor influencing PFS of TKI treatment in non-Xuanwei area patients (HR:1.760, 95% CI 1.106 to 2.800, *P*=0.017), and age (HR:1.501, 95% CI 1.158 to 1.946, *P*=0.002) and BMI (HR:0.621,95% CI 0.456 to 0.848, *P*=0.003) were independent factors affecting OS of TKI treatment in non-Xuanwei patients.

**Table 6:** Progress free survival and overall survival: univariate analysis of Non-Xuanwei lung cancer patients.

Parameters	Number	PFS(95%CI,month)	Chi-square	*P*	OS(95%CI,month)	Chi-square	*P*
**Gender**			3.574	0.059		3.801	0.050
Male	166	9.30(7.44-11.16)			25.50(20.28-30.72)		
Female	201	12.00(10.35-13.65)			28.90(22.29-35.51)		
**Ages**			1.424	0.233		9.799	**0.002**
<60	201	11.30(9.68-12.93)			32.70(28.70-36.70)		
≥60	166	9.50(8.28-10.72)			20.20(15.24-25.16)		
**Nationality**			1.369	0.242		0.009	0.924
Han	292	11.60(9.73-13.47)			26.10(21.75-30.45)		
Minority	75	9.20(7.69-10.71)			27.50(18.31-36.70)		
**BMI,kg/m^2^**			1.542	0.214		9.447	**0.002**
<24kg/m^2^	266	10.50(8.80-12.21)			23.30(20.00-26.60)		
≥24kg/m^2^	101	11.20(8.81-13.59)			39.50(30.51-48.49)		
**Smoking history**			0.08	0.777			
Smoker	113	9.20(6.76-11.64)			27.70(22.94-32.46)	0.896	0.344
Never-smoker	254	11.20(9.59-12.81)			25.10(19.47-30.73)		
**Drinking history**			1.561	0.212			
Yes	89	9.10(5.93-12.27)			25.60(16.50-34.70)	0.308	0.579
No	278	11.20(9.63-12.77)			27.00(22.36-31.65)		
**Hypertension history**			2.136	0.144		0.628	0.428
Yes	82	12.20(8.56-15.84)			29.20(17.87-40.53)		
No	285	10.40(8.88-11.93)			25.70(21.34-30.06)		
**Diabetes history**			1.513	0.219		3.218	0.073
Yes	15	7.60(2.24-12.96)			14.90(4.87-24.93)		
No	322	10.90(9.41-12.39)			27.40(23.26-31.54)		
**Lung cancer family history**			0.805	0.369		2.467	0.116
Yes	7	6.80(4.75-8.85)			18.00(0.00-39.48)		
No	360	10.70(9.41-12.19)			27.00(22.81-31.19)		
**Types of specimen**			2.089	0.148		1.495	0.222
Tissue	200	11.10(8.96-13.24)			28.80(23.77-33.83)		
Peripheral blood and pleural effusion	167	10.50(8.53-12.46)			25.40(20.30-30.51)		
**Clinical Stage**			0.011	0.915		0.254	0.615
IIIb	33	11.10(8.10-14.10)			34.30(19.79-48.81)		
IV	334	10.70(9.18-12.22)			26.10(22.15-30.05)		
**Brain metastasis**			1.277	0.259		1.375	0.241
Yes	107	9.10(7.41-10.79)			23.50(19.38-27.63)		
No	260	11.30(9.75-12.85)			30.30(25.40-35.20)		
**Drugs**			3.481	0.626		8.674	0.123
Gefitinib	212	11.20(9.54-12.86)			27.50(21.01-33.99)		
Icotinib	132	9.40(7.18-11.62)			27.40(22.15-32.65)		
Erlotinib	6	12.50(9.86-15.14)			25.40(NR)		
Afatinib	4	9.10(0.00-25.70)			22.90(5.98-39.82)		
Osimertinib	5	8.70(5.48-11.92)			31.90(7.411-56.39)		
unknown	8	7.40(0.00-17.77)			9.10(5.66-12.54)		
**Types of mutation**			5.872	**0.015**		1.266	0.268
classical mutation	347	11.10(9.59-12.61)			28.80(25.03-32.57)		
Uncommon mutation	20	5.10(0.00-11.46)			19.10(13.97-24.23)		

Bold values provided in **Tables 3**–**7** represents a p-value of less than 0.05, which is statistically different.

**Table 7:** Progress free survival and overall survival: univariate and multivariate analysis in Non-Xuanwei lung cancer subgroup.

Parameters	PFS				OS			
	Univariate		Multivariate		Univariate		Multivariate	
	HR(95%CI)	*P value*	HR(95%CI)	*P value*	HR(95%CI)	*P value*	HR(95%CI)	*P value*
Gender(Female)	0.813(0.655-1.009)	0.060			0.773(0.596-1.002)	0.052		
Age(≥60)	1.189(0.957-1.476)	0.117			1.508(1.164-1.955)	**0.002**	1.501(1.158-1.946)	**0.002**
Nationality(Minority)	1.170(0.898-1.525)	0.244			0.984(0.706-1.372)	0.924		
BMI(≥24kg/m^2^)	0.858(0.672-1.094)	0.216			0.618(0.453-0.843)	**0.002**	0.621(0.456-0.848)	**0.003**
Smoking history(Smoker)	1.034(0.818-1.307)	0.778			1.143(0.866-1.508)	0.345		
Drinking history(Yes)	1.171(0.913-1.501)	0.214			1.087(0.808-1.463)	0.579		
Hypertension history(Yes)	0.825(0.637-1.069)	0.146			0.882(0.647-1.203)	0.429		
Diabetes history(Yes)	1.454(0.796-2.654)	0.223			1.824(0.936-3.554)	0.078		
Lung cancer family history(Yes)	1.406(0.664-2.980)	0.373			1.897(0.841-4.278)	0.123		
Types of specimen(Tissue)	0.853(0.688-1.059)	0.150			0.850(0.655-1.103)	0.223		
Clinical Stage(IV)	0.995(0.908-1.090)	0.995			1.031(0.914-1.163)	0.615		
Brain metastasis(Yes)	1.144(0.905-1.446)	0.260			1.181(0.894-1.560)	0.242		
Types of mutation(Uncommon mutation)	1.760(1.106-2.800)	**0.017**	1.760(1.106-2.800)	**0.017**	1.490(0.895-2.479)	0.125		

Bold values provided in **Tables 3**–**7** represents a p-value of less than 0.05, which is statistically different.

As shown in **Table 6**, compared with the other study, our study had much longer PFS and OS in patients with EGFR uncommon mutation, especially in the Xuanwei subgroup, but had a little difference in PFS and OS in typical mutation patients.

**Figure 9:**
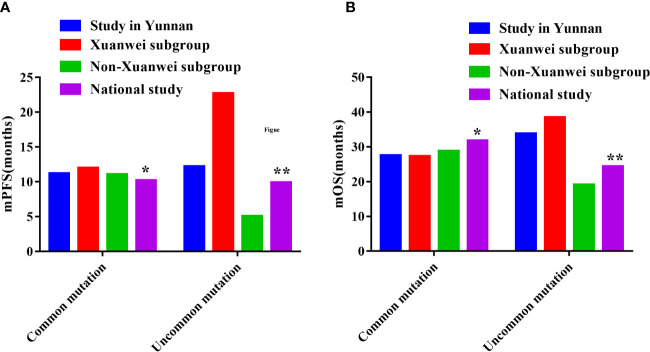
Comparisons of PFS and OS among the study in Yunnan, Xuanwei subgroup and other national muticenter study. **(A)** Comparisons of PFS. **(B)** Comparisons of OS. *FLAURA Study: Overall Survival with Osimertinib in Untreated, EGFR-Mutated Advanced NSCLC (9) ** UpSwing Study: A Retrospective International Cohort Study (UpSwinG) (10).

Compared with the FLAURA study, patients with EGFR classical mutation in our study had a little longer in PFS(11.20m vs. 10.20m) and a little shorter in OS(27.50m vs. 31.80m), the same as Xuanwei county subgroup (12.00m vs. 10.20m) and (27.30m vs. 31.80m), **Table 8** (9). Compared with the national multicenter real-world study of UpSwinG, we had a longer PFS (12.20m vs. 10.70m) and much longer OS(33.80m vs. 25.60m) in overall objectives with EGFR uncommon mutation (10). Again, in the Xuanwei subgroup, there was a much longer PFS(22.70m vs. 10.70m) and OS(38.50m vs. 25.60m), but there were shorter PFS(5.10m vs. 10.70m) and OS(19.10m vs. 25.60m) in Non-Xuanwei subgroup, **Table 8**, **Figure 9**.

**Table 8:** Comparisons of PFS and OS among the study in Yunnan, Xuanwei subgroup and other national muticenter study.

Parameters	mPFS (m,95%CI)				mOS (m,95%CI)			
	Study in Yunnan	Xuanwei subgroup	Non-Xuanwei subgroup	national study	Study in Yunnan	Xuanwei subgroup	Non-Xuanwei subgroup	national study
Types of mutation								
Common mutation	11.20 (9.97-12.43)	12.00 (9.29-14.71)	11.10 (9.59-12.61)	^a^10.20 (9.60-11.1)	27.50 (23.79-31.23)	27.30 (15.60-39.00)	28.80 (25.03-32.57)	^a^31.80 (26.60-36.00)
Uncommon mutation	12.20 (7.96-16.44)	22.70 (9.03-36.37)	5.10 (0.00-11.46)	^b^10.70 (9.2–12.9)	33.80 (27.21-40.39)	38.50(NR)	19.10 (13.97-24.23)	^b^25.60 (21.4–31.9)

a. FLAURA Study: Overall Survival with Osimertinib in Untreated, EGFR-Mutated Advanced NSCLC^[9]^.

b. UpSwinG Study:A Retrospective International Cohort Study (UpSwinG)^[10]^.

## Discussion

As the incidence rate and mortality of lung cancer in Xuanwei, YunnanProvince, China, is higher than that in the whole country and the rest the world, andseveral previous studies have demonstrated the genetic mutation characteristics oflung cancer patients with rare EGFR mutations, compound mutation rates, and RASmutation rates in Xuanwei (4, 5, 8). Therefore, conducting a comprehensive study onthe TKI treatment results in this region may provide a clinical basis forpersonalized clinical precision treatment of rare EGFR mutations in specific cancerhigh-risk areas and even other regions of the world.

Previous studies have suggested that EGFR-TKI is significantly more effectivein treating classical mutations than rare mutations (11, 12). In contrast, our overallstudy population showed no significant difference in efficacy between the twogroups.It is worth noting that univariate analysis revealed differences in PFSbetween regions, with Xuanwei lung cancer patients achieving longer mPFS thannon-Xuanwei lung cancer patients. However, analysis of patients in the Xuanweisubgroup showed that, in contrast, patients with rare mutations had better outcomesthan those with common mutations (5, 13). This may be related to the different typesand proportions of rare mutations in patients from different regions in each subgroup.Our previous studies showed that among rare mutations, the mutation rates ofEGFR-sensitive mutations such as G719X, G719X+L861Q, G719X+S768I, andS768I in Xuanwei were significantly higher than that in non-Xuanweiregions (13–15).

In addition, we compared the differences in PFS and OS between patients in ourstudy with FLAURA (9). It is difficult for us to make original comparison withoutthe raw data so a direct comparison between them was conducted. Generallyspeaking, the difference in PFS was not significant (11.2 m vs 10.2 m), while thedifference in OS was so apparent (27.5 m vs 31.8 m), with an averaged reduction of4.3 months. Previous in vitro studies found that the 19Del mutation has a higheraffinity for EGFR-TKI and thus may have a better effect on downstream signaling,while the 21 L858R mutation has a relatively low affinity for EGFR TKI and may beslightly less selective for EGFR TKI (15). Clinical study also discovered that patientswith 19Del mutation have better efficacy and better PFS and OS when treated withEGFR TKI compared to patients with 21 L858R mutation (16, 17). In our study,56.49% (235/416) of patients with 19-Del and 43.51% (207/416) of patients withL858R were included , while FLAURA study included 62.77% (349/556) and37.23% (207/556) accordingly, which possibly result in the different PFS. In recentyears, investigators have also explored the efficacy and prognosis of EGFR-TKItreatment for each 19Del subtype and found that different EGFR-TKI treatmentefficacy in patients with different Del- 19 subtypes was associated with differentsurvival, with longer PFS and OS for del E746 compared with del E746-A750, so wespeculate that there may be 19Del subtype differences or some specific unknownmutant loci to be further investigated in depth subsequently (18).

Regarding the EGFR rare mutation study population, the EGFR rare mutationpopulation in this study achieved longer PFS and OS compared to the globalUpSwinG multicenter study (10), with a prolonged mPFS of 1.5 months and aprolonged mOS of 8.2 months. The UpSwinG multicenter study included 246patients from 9 countries and regions worldwide, of which 83.7% were Asian and9.3% were Caucasian; the rare mutation types were common rare mutations (G719X,L861Q, S768I) accounting for 72.8%, and compound rare mutations accounting for32.8%. The majority of compound rare mutations were combinations of major raremutations. Interestingly, the UpSwinG study included subjects with similar raremutation types as the present study, with the difference that the proportion ofcompound rare mutations was higher in our study (59.6%), especially the highestproportion of patients with rare compound mutations in Xuanwei region. It is wellacknowledged that patients with compound mutations have improved outcome (13, 14), which may also be the potent reason for the longer mPFS and mOS obtainedin our study one of the most important reasons.

With the promotion of liquid biopsy in genetic testing, more and more studieshave confirmed that liquid biopsy can be an important complement to tissue biopsyin molecular testing. Studies clearly indicate that the positive detection rate of liquidbiopsy in EGFR gene testing is significantly lower than that of tissue samples, butstudies on whether there is a difference in the effect of subsequent TKI treatment indifferent sample testing populations are still lacking (5, 19). Our study suggests thatthe mPFS and mOS for EGFR-TKI therapy is different between different sampletypes, and the prognosis of the tissue sample delivery population is significantlybetter than that of the liquid biopsy and pleural effusion cytology deliverypopulations. However, a retrospective study (20) that included 59 samples showed nodifference in PFS and OS between patients with blood-delivered samples and thosewith tissue-delivered samples, which may be related to the small sample size and thehigh number of censored data described in the discussion of that study. We speculatethat this may be related to some differences in the accuracy of detection of fluid andplasma cavity effusion cytology specimens versus tissue samples. Interestingly, astudy that predicted the risk of TKI resistance by detecting EGFR mutations inplasma samples before and after TKI treatment suggested that a high rate of EGFRmutations was detected in the resistant patient population before clinical evaluationof resistance, which may suggest a relationship between plasma EGFR mutationsand TKI efficacy and resistance, but further studies are needed.

The NEJ002 study suggested that gefitinib, the first-generation TKI, was lesseffective than common mutation region in treating rare mutations in EGFR (21). However, a post hoc analysis of the LUX-Lung2, LUX-Lung3, and LUX-Lung6clinical studies showed that afatinib, the second-generation TKI yielded relativelygood data in patients with rare mutations such as L861Q, G719X, and S768I, with aPFS of up to 13.8 months and an OS of 26.9 months, with a sensitivity similar to thatof the common EGFR mutations, but its study sample size was only 75 cases (22). The German nNGM real-world study included 856 NSCLC cases with atypicalEGFR mutations (including co-mutations) from 12 centers and clinical follow-updata from 260 patients treated with different EGFR-TKI, chemotherapy, and immunecheckpoint inhibitors showed that patients with predominantly rare EGFR mutations(G719X, S7681, L861Q and above modifications coexisted) patients were treatedwith TKI, and 88.68% (415/468) of patients in this study population had a PFS of12.2 months with a generation TKI, suggesting that patients with rare mutations inthis region can benefit from the first generation TKI, and the benefit was moresignificant in the Xuanwei subgroup (23). This study will provide evidence-basedtreatment options for patients with rare EGFR mutations worldwide.

This study has its advantages. Sample size has always been difficult in raremutation population studies. Yunnan, especially Xuanwei, has a high rate of rareEGFR mutations and is an advantageous region for studying rare mutations. TheFLAURA study showed that the use of triple TKI axitinib in the first-line treatmentof advanced EGFR mutated NSCLC can achieve longer PFS and OS (24), andclinical guidelines have been approved as a Class IA evidence level 1recommendation and included in health insurance reimbursement (25). Unfortunately,however, patients with rare mutations have not yet been included in clinical trialstudies. It is also worthwhile to expect whether the third generation TKI can achievebetter efficacy in patients with rare EGFR mutations. The advantage of our study isthe inclusion of patients with rare mutations.

However, this study also had some limitations. First, this was a retrospectivestudy that includes only data from a single center. In addition, PFS survival datawere mature in this study. Still, the overall survival analysis outcome event has notyet reached more than 80%, which is 59.83%, which may be one of the reasons whysome variables in OS analysis (e.g, declared versus non-declared regions, classicalversus rare mutations, single versus compound mutations, and subgroup analysis)showed a trend of difference in values but did not reach statistical difference.Nevertheless, our study showed expected results that have not been reported before.We will continue to follow up and unveil the findings ofOS maturity data as soon aspossible.

In conclusion, this study reported the prognosis of EGFR-TKI treatment forNSCLC patients with different EGFR mutation types in Yunnan firstly, providingnew clinical evidence for EGFR-TKI-targeted therapy in patients with rare EGFRmutations in this region and worldwide. Prospective multicenter clinical studies areneeded to validate these observations, and further clinical studies are required. Welook forward to the participation of interested researchers from all over the world.We are also pleased to contribute more rare mutation cases from our region to otherresearch centers.

## Conclusion

NSCLC patients in Yunnan displayed a unique EGFR mutation profile, especially a higher prevalence of EGFR uncommon and compound mutations subtype. This study indicates prognostic factors of NSCLC treated with EGFR-TKI in Yunan and Xuanwei. This study will provide new clinical evidence for EGFR-TKI-targeted therapy in patients with rare EGFR mutations in China and worldwide. More research is needed for NSCLC EGFR-TKI therapy and medical insurance policy-making in Yunnan, Xuanwei area and uncommon especially.

## Data availability statement

The original contributions presented in the study are included in the article/**Supplementary Material**. Further inquiries can be directed to the corresponding authors.

## Author contributions

YPL, LC and RL contributed to conception and design of the study. XL, QL, JC, YD, GZ, XW, ZS, YDL and YC contributed to the acquisition, analysis, or interpretation of data for the work. YPL wrote the first draft of the manuscript. Then, LX, YZ and YH critically revised this report. All authors contributed to the article and approved the submitted version.
