# Supplementary figures and images for: Survival analysis of patients with advanced non-small cell lung cancer receiving EGFR-TKI treatment of Yunnan in southwestern China: a real-world study
Source: Front Oncol. 2023 Oct 10;13:1156647. doi: 10.3389/fonc.2023.1156647 (PMC10597689; doi:10.3389/fonc.2023.1156647)

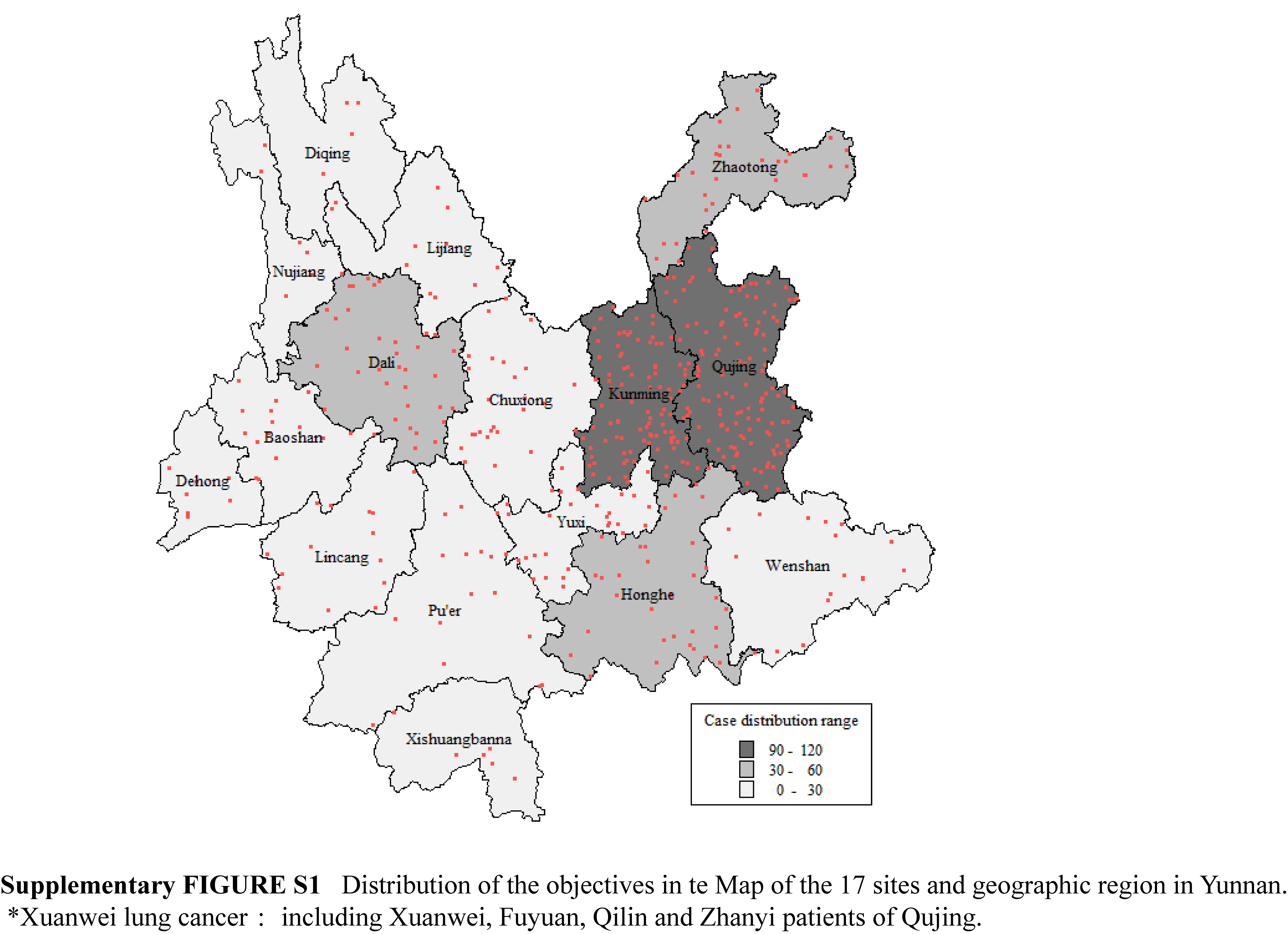

Supplement: Supplementary file 1 [file Image_1.jpeg]
